# Supplementary material for: Novel Driver Strength Index highlights important cancer genes in TCGA PanCanAtlas patients
Source: PeerJ. 2022 Aug 11;10:e13860. doi: 10.7717/peerj.13860 (PMC9375969; doi:10.7717/peerj.13860)
Supplement: Supplemental Information 2 [file peerj-10-13860-s002.zip › COHORTS/genes plots/cohorts/2021_8_16_14_9_distribution_cohorts_OV.pdf]

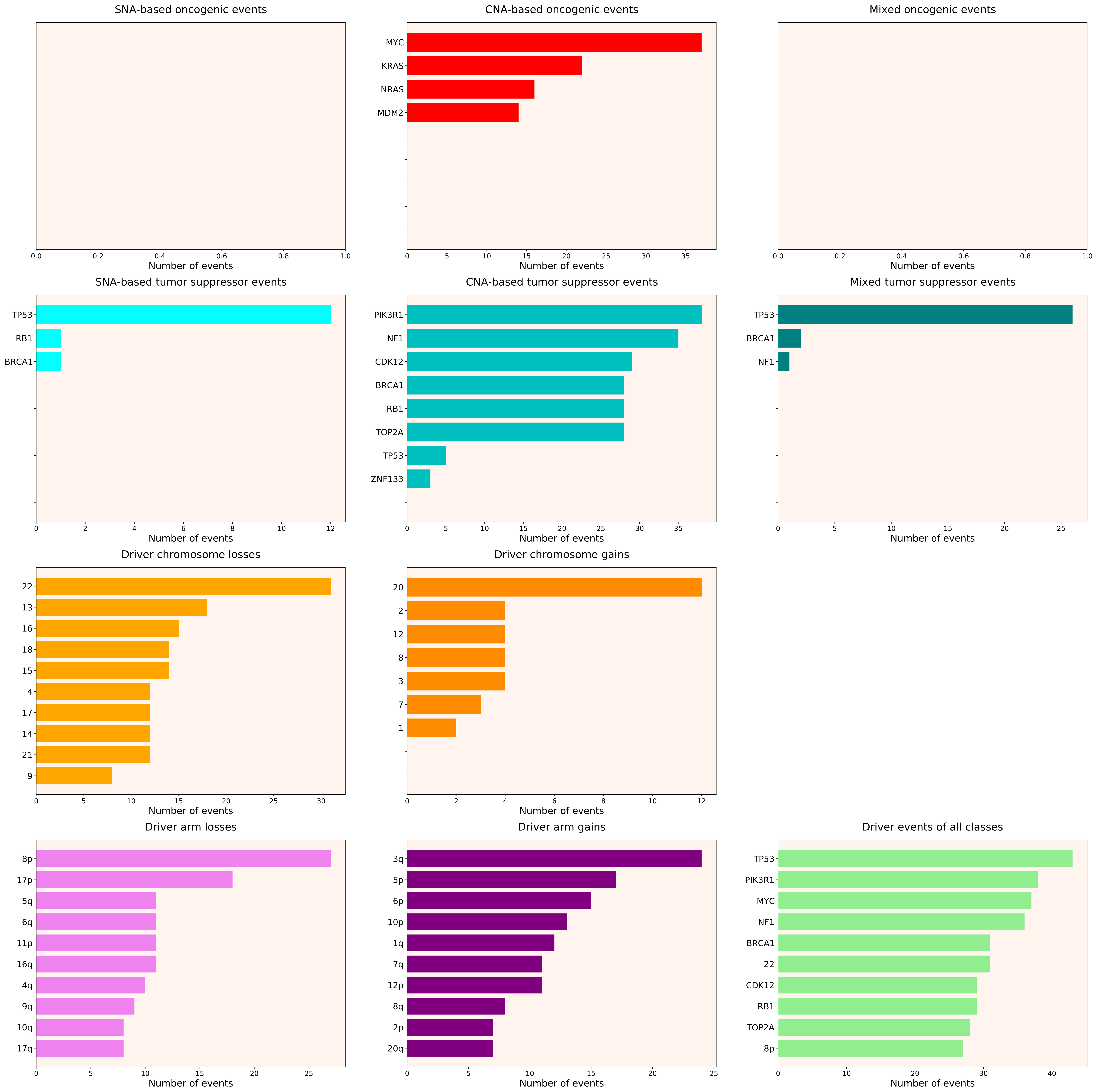
SNA-based oncogenic events
Number of events
CNA-based oncogenic events
MYC
KRAS
NRAS
MDM2
Number of events
Mixed oncogenic events
Number of events
SNA-based tumor suppressor events
TP53
RB1
BRCA1
Number of events
CNA-based tumor suppressor events
PIK3R1
NF1
CDK12
BRCA1
RB1
TOP2A
TP53
ZNF133
Number of events
Mixed tumor suppressor events
TP53
BRCA1
NF1
Number of events
Driver chromosome losses
22
13
16
18
15
4
17
14
21
9
Number of events
Driver chromosome gains
20
2
12
8
3
7
1
Number of events
Driver arm losses
8p
17p
5q
6q
11p
16q
4q
9q
10q
17q
Number of events
Driver arm gains
3q
5p
6p
10p
1q
7q
12p
8q
2p
20q
Number of events
Driver events of all classes
TP53
PIK3R1
MYC
NF1
BRCA1
22
CDK12
RB1
TOP2A
8p
Number of events
